# The cyclin-dependent kinase inhibitor 5, 6-dichloro-1-beta-D-ribofuranosylbenzimidazole induces nongenotoxic, DNA replication-independent apoptosis of normal and leukemic cells, regardless of their p53 status

**DOI:** 10.1186/1471-2407-9-281

**Published:** 2009-08-12

**Authors:** Valentina Turinetto, Paola Porcedda, Luca Orlando, Mario De Marchi, Antonio Amoroso, Claudia Giachino

**Affiliations:** 1 Department of Clinical and Biological Sciences, University of Turin, Regione Gonzole 10, 10043 Orbassano, Italy; 2 Department of Genetics Biology and Biochemistry, University of Turin, Via Santena, 19, 10126 Turin, Italy

## Abstract

**Background:**

Current chemotherapy of human cancers focuses on the DNA damage pathway to induce a p53-mediated cellular response leading to either G1 arrest or apoptosis. However, genotoxic treatments may induce mutations and translocations that result in secondary malignancies or recurrent disease. In addition, about 50% of human cancers are associated with mutations in the *p53 *gene. Nongenotoxic activation of apoptosis by targeting specific molecular pathways thus provides an attractive therapeutic approach.

**Methods:**

Normal and leukemic cells were evaluated for their sensitivity to 5, 6-dichloro-1-beta-D-ribofuranosylbenzimidazole (DRB) through cell viability and caspase activation tests. The apoptotic pathway induced by DRB was analysed by immunfluorescence and immunoblot analysis. H2AX phosphorylation and cell cycle analysis were performed to study the dependance of apoptosis on DNA damage and DNA replication, respectively. To investigate the role of p53 in DRB-induced apoptosis, specific p53 inhibitors were used. Statistical analysis on cell survival was performed with the test of independence.

**Results:**

Here we report that DRB, an inhibitor of the transcriptional cyclin-dependent kinases (CDKs) 7 and 9, triggers DNA replication-independent apoptosis in normal and leukemic human cells regardless of their p53 status and without inducing DNA damage. Our data indicate that (i) in p53-competent cells, apoptosis induced by DRB relies on a cytosolic accumulation of p53 and subsequent Bax activation, (ii) in the absence of p53, it may rely on p73, and (iii) it is independent of ATM and NBS1 proteins. Notably, even apoptosis-resistant leukemic cells such as Raji were sensitive to DRB.

**Conclusion:**

Our results indicate that DRB represents a potentially useful cancer chemotherapeutic strategy that employs both the p53-dependent and -independent apoptotic pathways without inducing genotoxic stress, thereby decreasing the risk of secondary malignancies.

## Background

Current chemotherapy focuses on the use of genotoxic drugs. This may induce general DNA damage in cancer cells but also high levels of toxicity in normal tissues. Reports over the last 10 years have described new, therapy-related, malignancies whose prognosis is often poor due to resistance [1-4]. Most cytotoxic drugs, and radiotherapy, damage tumour cell DNA to induce arrest in G1 or apoptosis [5,6]. However, DNA damage is also induced in normal cells. It has been shown that alkylating agents and cisplatin cause unbalanced chromosomal aberrations [7], and epipodophyllotoxins (inhibitors of topoisomerase II) have been implicated in translocations involving chromosome bands 11q23 and 21q22, both of which are associated with secondary malignancies [3,8,9]. In addition, most chemotherapy treatments rely on induction of p53-dependent apoptosis. The efficiency of this approach, however, is diminished by the fact that the *p53 *gene is mutated in about 50% of human cancers. Moreover, it is becoming clear that a high percentage of resistant and recurrent tumours carry *de novo p53 *mutations [2,4,6,10].

Nongenotoxic activation of apoptosis by targeting specific molecular pathways therefore provides an attractive therapeutic strategy in cancers. Inhibition of transcription induces apoptosis in several cancer cell lines [11,12], and this apoptosis may be more pronounced in transformed cells than in their non-transformed counterparts [13]. One class of transcriptional inhibitors comprises the inhibitors of the CDKs, whose critical role in cell cycle progression and cellular transcription make them attractive targets for the elaboration of new anticancer drugs [14]. A few inhibitors of transcriptional CDKs, including flavopiridol and seliciclib, are currently providing encouraging results in clinical trials, though some pharmacokinetic concerns remain to be solved and some aspects of the biological response they elicit are still undetermined [14-17]. It is thus of interest to further evaluate the biological effects and potential anti-cancer role of inhibitors of transcriptional CDKs.

DRB is a potent inhibitor of CDK7 and CDK9, kinases that phosphorylate the COOH-terminal domain (CTD) of the largest subunit of RNA polymerase II (pol II) [18,19]. It inhibits more than 50% mRNA synthesis at doses above 40 μM [13,20] and has been shown to inhibit both transcription *in vivo *[21] and phosphorylation of the pol II CTD *in vitro *[18]. Additionally, DRB also inhibits other protein kinases involved in cellular metabolism such as casein kinase type I (CK1) and II (CK2) [22]. Blockade of pol II-dependent transcription, including that elicited by DRB, had been shown previously to trigger a cell death signal [11,13,20,23]. The exact underlying mechanisms, however, are still unclear, particularly with respect to the question of p53-dependence and the need for ongoing DNA replication.

In the present study we demonstrate that DRB is highly cytotoxic regardless of a cell's p53 status and even in the absence of active DNA synthesis. Prototypic T-, B- and myelogenous leukaemia cell lines as well as fresh AML blasts were all susceptible to DRB-induced apoptosis. Our results suggest that DRB could be an attractive drug for further evaluation in the treatment of some forms of cancer.

## Methods

### Cell isolation, cell culture and drug treatment

Peripheral blood from four healthy donors, one AT and one NBS patients was collected after signed informed consent. The AT patient was homozygous for a truncating mutation; the NBS patient was homozygous for the common 657del5 mutation. PBMC were isolated and T cell lines, generated as described [24], were maintained by periodic stimulation with PHA (Gibco-Invitrogen, Paisley, UK) and irradiated allogeneic PBMC in complete RPMI medium (RPMI 1640 added with 1% Kanamycin, 1% Sodium Pyruvate, 1% L-Glutamine, 1% non essential amino acids, 0,1% β-mercapto-ethanol) (all from Gibco-Invitrogen), supplemented with 5% human serum (BioWitthaker, Cambrex, Baltimore, MD, USA) and 200 U/ml recombinant IL2 (from the myeloma producing cell line IL2-t6, kindly provided by Dr A. Lanzavecchia, IRB, Bellinzona, Switzerland). LCLs were generated by transformation of mononuclear cells with Epstein-Barr virus and maintained at 37°C in a humidified incubator in the presence of 5% CO2 in complete RPMI medium supplemented with 10% heat-inactivated fetal bovine serum (Gibco-Invitrogen). The human leukaemia cell lines Jurkat and MOLT-4, of lymphoid origin, HL60, of myeloid origin, and Namalwa and Raji from B-cell Burkitt's lymphomas were purchased from the American Type Culture Collection (Rockville, MD, USA) and grown in complete RPMI medium supplemented with 10% heat-inactivated fetal bovine serum (Gibco, Invitrogen). One bone marrow acute myeloid leukaemia (AML) sample was collected at San Luigi's Hospital after signed informed consent. Mononuclear cells were separated by Ficoll-Paque density centrifugation (Amersham, GE Healthcare, Buckinghamshhire, UK). Primary AML cells were kept in complete RPMI medium supplemented with 10% heat-inactivated fetal bovine serum (Gibco, Invitrogen). Leukemic blast cell count in the primary sample was about 60%. TAp63α transfected 293T cells were kindly provided by Dr M. Lo Iacono, University of Turin, Italy. The transcription inhibitor DRB (Sigma-Aldrich Co., St. Louis, MO, USA) was used at 10–100 μM. Z-VAD-FMK peptide was obtained from Promega (Madison, WI, USA) and added at 100 μM at the same time that apoptosis was induced. α-PFT and μ-PFT (Sigma-Aldrich Co.) were used at 30 μM and 10 μM, respectively, and added 20 minutes before DRB. Cultured T cells were γ-irradiated (2 Gy) using a 6 MV accelerator (Elekta) at a dose of 2 Gy/min. ActD supplied by Sigma-Aldrich Co. was used at the dose of 0.05 μg/ml.

### Flow cytometry

The cell viability was determined by PI (Sigma-Aldrich Co.) staining; PI was used at the final concentration of 1 μg/ml and incubated at room temperature for 15 min in the dark before the analysis. Cell survival was expressed after normalization to medium supplemented with the drug's solvent ("medium" in the graphs) and shown as mean ± S.D Caspase activation was analyzed with anti-Active Caspase-3 (BD PharMingen, San Diego, CA, USA) as primary antibody and a PE-conjugated goat anti-rabbit (BD PharMingen) as secondary antibody and using the Cytofix/Cytoperm Kit (BD PharMingen). Annexin V-FITC (BD PharMingen) and PI staining was performed in accordance with the manufacturer's instructions. CD45 staining was performed with anti- CD45-FITC antibody (BD PharMingen). Cell cycle analysis was based on DNA content. Briefly, ethanol-fixed cells were treated with 1 μg/ml RNaseA (Sigma-Aldrich Co.) and stained with 50 μg/ml PI. Cells were plotted in a FL2W (width) versus FL2A (area) dot plot and gated to exclude aggregates; gated cells were plotted in a FL2A histogram to distinguish the cell cycle phases. To select circulating lymphocytes within PBMC, lymphocytes were gated based on the SSC and FSC parameters. Stained cells were analysed on a FACScan (Becton Dikinson & Co., San Jose CA, USA). Statistical analysis on cell survival was performed with the test of independence.

### Immunofluorescence

Approximately 400,000 T cells for each condition were collected, fixed with 4% paraformaldehyde, permeabilized with 0.5% Triton X-100, and blocked with 6% bovine serum albumin and 2.5% normal goat serum. They were stained with anti- phospho-Histone H2AX (Ser139) (Upstate Biotechnology, Charlottesville, VA, USA), anti-p53 or anti-BAX (Santa Cruz Biotechnology, Santa Cruz, CA, USA) specific Abs, and with Alexa 546-conjugated goat anti-mouse as secondary Ab (Molecular Probes, Invitrogen). MitoTraker Green FM (Molecular Probes, Invitrogen) was used at the final concentration of 100 nM. Stained cells were transferred to poly-L-lysine-coated coverslips and slides were mounted with Mowiol (Calbiochem, San Diego, CA, USA). Fluorescence images were obtained with a 510 Carl Zeiss confocal laser microscope using a 63× objective. Optical sections through the nuclei were captured at 0.5 μm intervals, and images were obtained by projection of the individual sections. For H2AX phosphorylation quantitative analysis, foci were counted by eye until at least 40 cells and 40 foci were recorded per sample.

### Cell extracts and subcellular fractionation

Cells were harvested and washed with PBS, pelleted, and lysed in Laemmli buffer (0.125 M Tris-HCl [pH 6.8], 5% SDS) containing as inhibitors 1 mM phenylmethylsulfonyl fluoride, pepstatin (10 μg/ml), aprotinin (100 KIU/ml), leupeptin (10 μg/ml) and 1 mM sodium orthovanadate (Na_3_VO_4_) (all from Calbiochem). Total lysates were boiled for 2 min, sonicated, and quantified by the micro-bicinchoninic acid method (Thermo Scientific, Rockford, IL, USA). Protein content was checked probing the blots with either vinculin or β-actin specific Abs. For subcellular fractionation, 2 × 10^7 ^T cells were harvested and washed with PBS; mitochondrial and cytosolic fractions were isolated with the use of the Mitochondria Isolation Kit for cultured cells (Thermo Scientific, reagent-based method). The mitochondrial pellet was lysed in Laemmli buffer, and the cytosolic supernatant was concentrated with a Microcon device (size cut-off 10 kDa) (Millipore Corporation, Bedford, MA, USA). Both fractions were quantified by the micro-bicinchoninic acid method (Thermo Scientific) before analysis by immunoblotting. Protein content and purity of the fractions were checked probing the blots with vinculin and TOM40 specific Abs.

### Immunoblot analysis

40 μg of total lysates and 20 μg of mitochondrial and cytosolic fractions were size fractionated by SDS-PAGE 7 to 10% gels and electroblotted onto polyvinylidene difluoride membranes (Amersham, GE Healthcare). After blocking with 5% non-fat dried milk in PBS plus 0.1% Tween (Sigma-Aldrich Co.), the membranes were incubated with anti -p53 (YLEM, Avezzano, Italy) or -p53-pSer15 (Cell Signaling Technology, Danvers, MA, USA.), -MDM2, -BAX, -TOM40, -p73 (Santa Cruz Biotechnology), -p63 (Biomeda, Foster City, CA, USA), -vinculin, -β-actin (Sigma-Aldrich Co.) specific Abs and subsequently with peroxidase-conjugated secondary antibodies (Amersham, GE Healthcare). The immunoreactive bands were visualized by ECL Super Signal (Thermo Scientific) on autoradiographic films. Autoradiographic bands were scanned and quantified by Kodak 1D Image Analysis Software.

## Results

### The stress response elicited by DRB in human lymphocytes results in DNA replication-independent, DNA damage-independent and p53-mediated apoptosis

**Figure 1:**
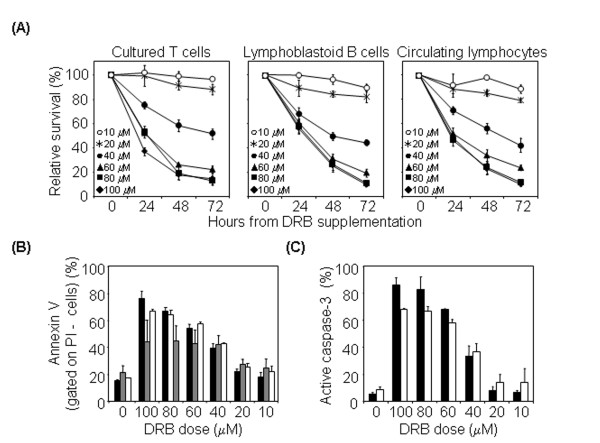
**DRB induces apoptotic death in T cell lines, LCLs and circulating lymphocytes**. (A) DRB induces lymphocytes death. Cultured T cells from four donors (left), LCLs from three donors (middle) and circulating lymphocytes from three donors (right) were kept in medium alone or containing 10–100 μM DRB, harvested at 24, 48 and 72 hr and analysed by flow cytometry after stainig with PI. (B) DRB treatment induces translocation of phosphatidylserine to the external leaflet of the cell membrane. Cultured T cells from two donors (black bars), circulating lymphocytes from two donors (white bars) and LCLs from two donors (grey bars) were kept for 24 hr in medium alone or containing 10–100 μM DRB, harvested and stained with annexin V and PI. Percentages of PI- annexin V+ cells are indicated (mean ± S.D.). (C) DRB treatment induces caspase activation. Cultured T cells from four donors (black bars) and circulating lymphocytes from three donors (white bars) were kept for 24 hr in medium alone or containing 10–100 μM DRB, harvested and stained with anti-active caspase-3 antibody. Histograms show the percentage of active caspase-3^+ ^cells (mean ± S.D.).

We investigated the effects induced by DRB in human cells. To define the sensitivity of cultured T lymphocytes to this drug, we assessed the viability of treated cells in a dose-response experiment using propidium iodide (PI) staining and flow cytometry. T lymphocytes were susceptible to DRB-induced death within the 40–100 μM range in a dose-dependent manner (mean 15% viable cells at 72 h with 100 μM DRB) (Figure 1A). The death mechanism was apoptosis, as both annexin V+PI- (i.e. early apoptotic cells, mean 76% at 24 hr with 100 μM DRB) and active caspase-3+ cells (mean 86% at 24 hr with 100 μM DRB) could readily be detected (Figure 1B–C). Analogous results were obtained with proliferating lymphoblastoid B cell lines (LCLs) (mean 10% viable cells at 72 hr with 100 μM DRB) and with freshly extracted circulating lymphocytes (mean 10% viable cells at 72 hr with 100 μM DRB) (Figure 1A–C). All cell lines showed significant sensitivity to DRB (p < 0.05 with the test of independence) at doses above 40 μM, compared to untreated cells.

**Figure 2:**
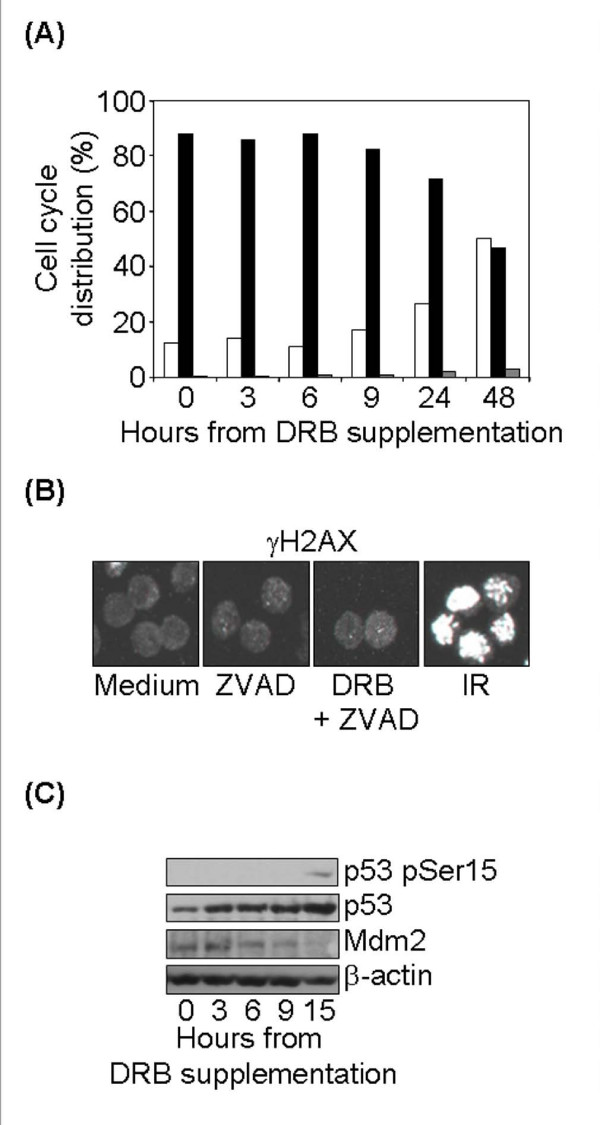
**DRB-induced apoptosis is replication-independent, DNA damage-independent and p53-mediated**. (A) DRB treatment does not induce entry into S phase. Circulating lymphocytes from one healthy donor were maintained in medium alone or containing 100 μM DRB, harvested 3, 6, 9, 24 and 48 hr after treatment, fixed and analysed for cell cycle. Histograms show the percentage of cells in subG0/G1 (white bars), in G0/G1 (black bars) or in S-G2/M (grey bars) phases of the cell cycle at each treatment time. (B) DRB treatment does not induce DSBs. Cultured T cells from one donor were maintained in medium alone, supplemented with the caspase inhibitor ZVAD or supplemented with 100 μM DRB and ZVAD. Part of the cells kept in medium alone were irradiated with 2 Gy γ-IR. Cells were harvested 24 hr after 100 μM DRB or 1 hr after 2 Gy γ-IR and stained for γ-H2AX. One representative experiment out of two is shown. (C) Immunoblot analysis of p53, p53pSer15 and Mdm2 following DRB. Time course analysis of normal T cells harvested before (0) or at various times after 100 μM DRB treatment. One representative experiment out of three is shown.

Many agents that block pol II elongation also block DNA replication. To rule out the possibility that apoptosis was induced exclusively upon entry of the cells into S phase, we determined the cell cycle profile of freshly extracted circulating lymphocytes, virtually all in the G0/G1 phase, at different time points after DRB treatment, and found that apoptosis occurred without passage through the S phase (Figure 2A). Comparable results were obtained with quiescent cultured T lymphocytes (data not shown).

To verify that DRB induced apoptosis was not attributable to induction of DNA damage, we assessed phosphorylation of the histone variant H2AX on serine 139 (γH2AX), an early and specific indicator of DNA double-strand breaks (DSBs) [25], by immunofluorescence and confocal microscopy. As apoptosis itself results in oligonucleosomal fragmentation of DNA and H2AX phosphorylation [26], these experiments were done in the presence of the caspase inhibitor ZVAD-FMK. Untreated cells displayed virtually no γH2AX foci (mean 0,83 ± 2,13 foci/cell); after DRB treatment γH2AX foci formation did not occur (mean 0,87 ± 2,01 foci/cell) (Figure 2B).

We then investigated the molecular pathway elicited by DRB in T lymphocytes. As shown in figure 2C, DRB rapidly stabilized p53, while phosphorylation of p53 at Ser15 was only detectable when p53 accumulation had already reached the high level. As phosphorylation of Ser15 on p53 is synonymous with the activation of DNA damage-dependent pathways in response to cellular insults [27], this provides further proof that no genotoxic stress is imposed on cells treated with DRB. p53 accumulation was not contrasted by Mdm2, a p53 target required for p53 degradation [28], as this protein was rapidly down regulated, possibly due to the known transcriptional inhibitory effect of DRB (Figure 2C).

Overall, these results indicate that the stress response elicited by DRB in human T cells results in replication- and DNA-damage-independent apoptosis. They also suggest that the death pathway thus induced is p53-mediated.

### Cytosolic p53 accumulates in pre-apoptotic DRB-treated T lymphocytes and correlates with Bax activation

**Figure 3:**
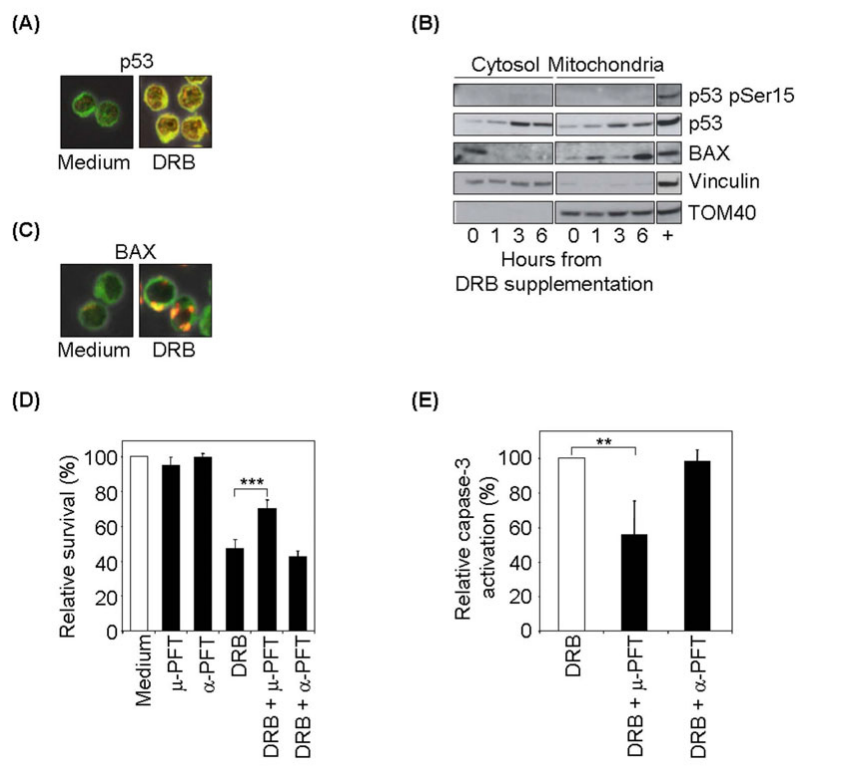
**DRB-induced apoptosis is mediated by cytosolic p53 accumulation and Bax activation**. (A) Confocal analysis of p53 localization. Control T cells were harvested before or 6 hr after 100 μM DRB treatment and stained for p53 and with green MitoTraker as cytosolic marker. One representative experiment out of three is shown. (B) Immunoblot analysis of p53, p53pSer15 and Bax localization. Time course analysis of cytosolic and mitochondrial fractions on control T cells after 100 μM DRB treatment. One representative experiment out of two is shown. Positive control (+ lane): whole cell lysate from a control T cell line treated for 6 hr with 0,05 μg/ml ActD. (C) Confocal analysis of Bax distribution. Control T cells were harvested before or 6 hr after 100 μM DRB and stained for Bax and with green MitoTraker as cytosolic marker. One representative experiment out of three is shown. (D) Survival analysis of DRB-treated T cells after p53 inhibition. Control T cells were treated with either α-PFT or μ-PFT alone or with 100 μM DRB and analyzed at 24 hr by flow cytometry after stainig with PI and (E) with anti-active caspase-3 antibody. Data are normalized to caspase activation with DRB alone (mean ± S.D.).

In the light of DRB's potent inhibition of transcription [13,18,20,21], we reasoned that the apoptotic pathway it induced did not rely on the transactivation activity of p53. Recent studies have linked the non-transcriptional pro-apoptotic activity of p53 to its cytosolic or mitochondrial localization [29,30]. To determine whether this activity contributes to DRB-induced apoptosis in human T cells, we monitored the p53 location of DRB-treated cells. We first analyzed p53 distribution by immunostaining and confocal microscopy. A time-course experiment shown that active caspase-3 becomes detectable 9 hr after DRB treatment (data not shown), indicating that the signals which initialize the apoptotic cascade must occur earlier. We therefore performed p53 confocal microscopy analysis of T lymphocytes harvested at 6 hr from treatment and evaluated its nuclear versus cytosolic localization. p53 was preferentially accumulated in the cytosol (Figure 3A). We then prepared cytosolic and mitochondrial fractions of T lymphocytes harvested at 1–3–6 hr from treatment, and determined the distribution of p53 with respect to its mitochondrial location. Purity of the fractions was checked by probing the blots for vinculin and TOM40 as markers for the cytosolic and mitochondrial compartments, respectively (Figure 3B). Results clearly demonstrated the presence of extranuclear p53 soon after DRB treatment; it was present in both the cytosolic and the mitochondrial fractions, and primarily unphosphorylated on Ser15 (Figure 3B).

Cytosolic accumulation of p53 has been reported to directly activate Bax to insert and oligomerize into the mitochondrial membrane, thus leading to the cascade of mitochondria-mediated apoptosis and caspase-3 activation [29]. We therefore investigated whether intrinsically generated high cytosolic p53 levels in DRB-treated cells were coupled to activation of Bax. We monitored Bax distribution by immunofluorescence and confocal microscopy. In untreated cells Bax appeared in a basal cytosolic distribution (Figure 3C), whereas 6 hr after DRB treatment a dot-like pattern was displayed by about 20% of cells and then by virtually 100% after 15 hr (Figure 3C and data not shown). To better evaluate Bax activation, we studied the distribution of endogenous Bax with respect to its mitochondrial localization by fractionation experiments. Results demonstrated that in untreated cells Bax was free in the cytosol, whereas during DRB treatment it progressively translocated to the mithocondria, where it was retained throughout the entire time-course (Figure 3B).

To further investigate the role of mitochondrial p53 in DRB-induced apoptosis we used pharmacological manipulations to distinguish nuclear and mitochondrial p53 functions. T cells were preincubated with two inhibitors of p53 that have different mechanisms of action: α-pifithrin (α-PFT) reversibly blocks the p53-mediated transactivation of p53-dependent responsive genes [31]; μ-pifithrin (μ-PFT) blocks the interaction of p53 with Bcl-xL and Bcl-2 and selectively inhibits p53 translocation to the mitochondria, without affecting the transactivation function of p53 at the level of the genome [32].

While the viability level of DRB-treated T cells did not change in the presence of α-PFT, preincubation with μ-PFT conferred partial resistance toward DRB induced death, with viability at 24 hours of 70% (p < 0,005 compared to DRB alone) (Figure 3D). To assess whether the blockage of mitochondrial p53 does increase viability through apoptosis inhibition we analysed caspase-3 activation in control T cells treated with DRB, either alone or in the presence of the two p53 inhibitors. We found that μ-PFT preincubation reduced caspase-3 activation of about 50% as compared to DRB alone (p < 0,05 compared to DRB alone) (Figure 3E). As expected, α-PFT treatment had no effect on caspase-3 activation.

These results indicate that DRB leads to p53 accumulation mainly in the mitochondria and cytosol, without inducing DNA damage. They also suggest that p53 induces Bax activation and this may be an initiation step of DRB-induced apoptosis.

### DRB-induced apoptosis is independent of ATM and NBS1

**Figure 4:**
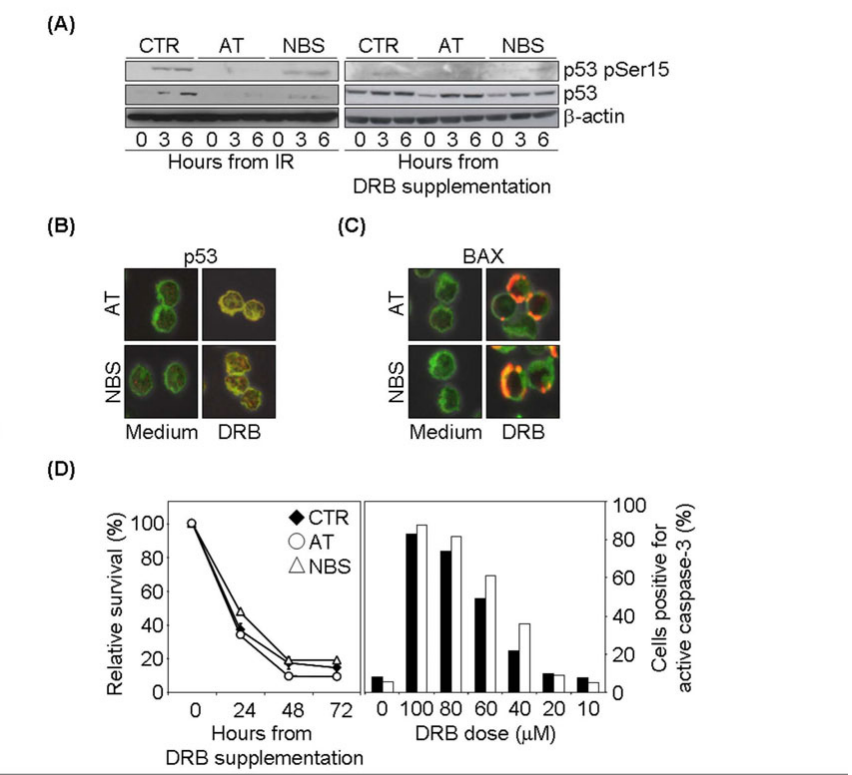
**DRB-induced apoptosis is independent of ATM and NBS1**. (A) Immunoblot analysis of p53 and p53pSer15 in AT and NBS cells. Time course analysis of whole cell lysates of cultured T cells from one control, one AT and one NBS patient harvested before (0) or after either 2 Gy γ-IR or 100 μM DRB treatment. One representative experiment out of three is shown. (B, C) Confocal analysis of p53 and Bax localization. Cultured T lymphocytes from one AT and one NBS patient were harvested before or 6 hr after 100 μM DRB and stained for either p53 or Bax with green MitoTraker as cytsolic marker. One representative experiment out of two is shown. (D) AT and NBS cell survival and caspase activation following DRB treatment. Left panel: cultured T cells from one AT, one NBS patient and four controls were kept in medium alone or containing 100 μM DRB, harvested at 24, 48 and 72 hr and analyzed by flow cytometry after staining with PI. Right panel: cultured T cells from one AT (black bars) and one NBS (white bars) patient were kept for 24 hr in medium alone or containing different concentrations of DRB, then harvested and stained with anti-active caspase-3 antibody. Histograms show the percentage of active caspase-3^+ ^cells.

T cells from Ataxia-Telangiectasia (AT) and Nijmegen Breakage Syndrome (NBS) patients are resistant to the p53-dependent apoptosis induced by DNA intercalating agents like Actinomycin D [33] and display a clear p53 accumulation defect following genotoxic stimuli such as ionizing radiation (IR) (Figure 4A). However, DRB-induced p53 accumulation was comparable to that of normal cells, as assessed by both western blot analysis and confocal microscopy (Figure 4A and 4B). As in normal T lymphocytes, accumulated p53 was essentially cytosolic (Figure 4B), and assessment of their Bax distribution revealed a dot-like pattern after DRB treatment, at levels comparable to the control cells (Figure 4C). Accordingly, AT and NBS T lymphocytes readily died after DRB treatment, with caspase-3 activation comparable to that of control T lymphocytes (Figure 4D). These results suggest that DRB-induced accumulation of p53 and subsequent apoptosis are ATM- and NBS1-independent.

### In the absence of p53, DRB-induced apoptosis is mediated by p73

**Figure 5:**
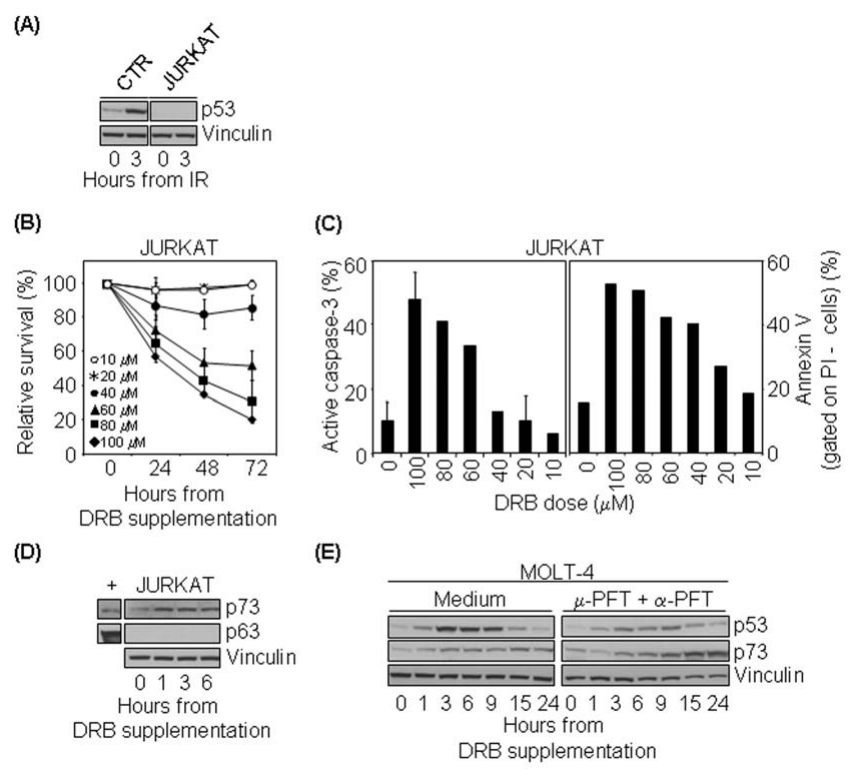
**In the absence of p53, DRB-induced apoptosis is mediated by p73**. (A) Analysis of p53 on Jurkat cells. Western blot analysis was performed on control and Jurkat T cells harvested before or 3 hr after 2 Gy γ-IR. (B) DRB induces Jurkat cell death. Jurkat cells were kept in medium alone or containing 10–100 μM DRB, harvested at 24, 48 and 72 hr and analyzed by flow cytometry after staining with PI. p < 0.05 with the test of independence at doses above 40 μM, compared to untreated cells. (C) DRB induces Jurkat apoptosis. Jurkat cells were harvested before or 24 hr after 10–100 μM DRB and stained with anti-active caspase-3 antibody (left panel) and with annexin V and PI (right panel). Histograms show the percentage of active caspase-3^+ ^cells (mean ± S.D.) and PI- annexin V+ cells. (D) Immunoblot analysis of p73 and p63 on Jurkat cells harvested before (0) or after 100 μM DRB. p73 and p63 positive controls (+ lane): whole cell lysate from HL60 and TAp63α transfected 293T cells, respectively. (E) Inhibition of p53 in the p53 wild-type tumour cell line MOLT-4 induces p73 activation. Western blot analysis of MOLT-4 cells harvested before (0) or after 100 μM DRB alone (left panel) or following preincubation with α-PFT and μ-PFT (right panel).

T-cell leukaemia Jurkat cell line lacks p53 protein, perhaps as the outcome of missense heterozygous point-mutations in the *p53 *gene [34,35]. It was therefore of interest to see whether Jurkat T cells undergo apoptosis following DRB treatment. Western blot analysis confirmed that p53 was not expressed in our Jurkat T cells, not even following a genotoxic treatment (2 Gy IR) known to induce high levels of p53 (Figure 5A). Nevertheless, they readily died after DRB in a dose-dependent manner (mean 20% viable cells at 72 hr with 100 μM DRB) (Figure 5B) and both active caspase-3 and surface annexin V expression were detected after treatment, though in a slightly smaller percentage of cells compared to control T lymphocytes (mean 48% and 52% of positive cells, respectively, at 24 hr with 100 μM DRB) (Figure 5C). Thus, DRB-induced apoptosis in these cells occurs despite the absence of p53.

To determine which alternative molecular pathway is induced by DRB in the absence of p53, we performed immunoblot analysis in Jurkat T cells for two p53 family members known to be involved in apoptosis, p63 and p73 [36]. As shown in figure 5D, the p73 content increased significantly in the total cell lysates within 1 hr of exposure to 100 μM DRB and remained upregulated throughout the time-course, whereas p63 protein was never detectable.

To further demonstrate the p73-mediated alternative pathway we pharmacologically inhibited p53 activity in the MOLT-4 p53 wild type leukemic cell line [37] and compared p73 induction with and without p53 blockage. In the absence of p53 inhibition, immunoblot analysis showed that DRB induced early p53 accumulation, comparable to that of normal T cells, and p73 content only minimally increased. When MOLT-4 cells were preincubated with the two p53 inhibitors, α-PFT and μ-PFT, p53 accumulation was reduced and p73 content increased significantly at later time points (Figure 5E).

These data suggest that DRB induces a redundant death pathway and that apoptosis may rely on p73 activation in the absence of p53.

### Prototypic T, B and myelogenous leukaemia/lymphomas are susceptible to DRB-induced apoptosis irrespective of their p53 status

Having shown that the Jurkat line is sensitive to DRB-induced apoptosis, we examined DRB's effects on cultured HL60 cells, a model of acute myelogenous leukaemia, and on Namalwa and Raji cells, two model Burkitt's lymphoma lines.

**Figure 6:**
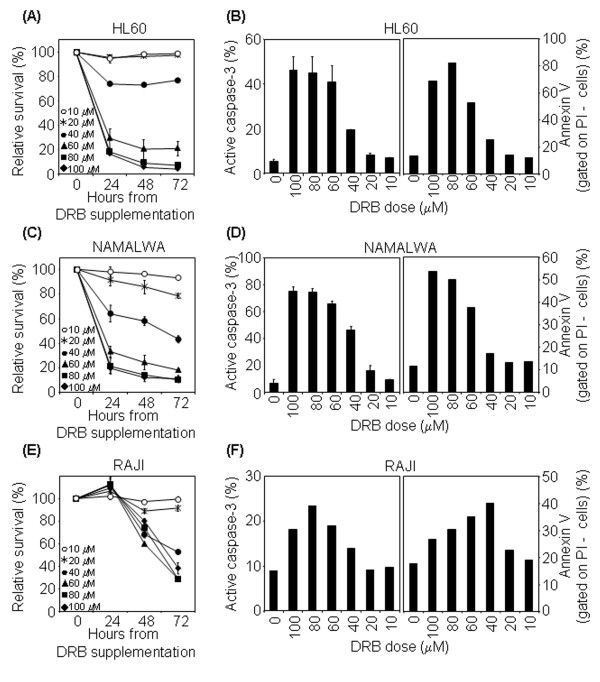
**Sensitivity to DRB-induced apoptosis in prototypic leukaemia/lymphoma cell lines**. (A) DRB induces HL60 cell death. HL60 cells were cultured in medium alone or containing 10–100 μM DRB, harvested at 24, 48 and 72 hr and analysed by flow cytometry after staining with PI. p < 0.005 with the test of independence at doses above 40 μM, compared to untreated cells. (B) DRB induces HL60 caspase activation and translocation of phosphatidylserine to the external leaflet of the cell membrane. Left panel: HL60 cells were kept for 24 hr in medium alone or containing 10–100 μM DRB, then harvested and stained with anti-active caspase-3 antibody. Histograms show the percentage of active caspase-3^+ ^cells (mean ± S.D. of two independent experiments). Right panel: HL60 cells were kept for 24 hr in medium alone or containing 10–100 μM DRB, harvested and stained with annexin V and PI. Percentages of PI- annexin V+ cells are indicated. (C, D) The same experiments were performed on Namalwa cells. (E, F) The same experiments were performed on Raji cells, analysing caspase activation and translocation of phosphatidylserine to the external leaflet of the cell membrane 48 hr after treatment.

In HL60 cells *p53 *gene underwent major deletions, so these cells completely lack *p53 *mRNA and protein [38] (and our data not shown). Nevertheless, they were highly susceptible to DRB-induced death within the 40–100 μM range (mean 4% viable cells at 72 hr with 100 μM DRB) (Figure 6A) and both active caspase-3 and surface annexin V expression were detected after treatment, though in a slightly smaller percentage of cells compared to control T lymphocytes (mean 46% and 69% of positive cells, respectively, at 24 hr with 100 μM DRB) (Figure 6B). It is noticeable that HL60 cells were more sensitive at 24 h than control cells.

The Namalwa and Raji lines are mutant for *p53*: Namalwa cells are *p53 *double-mutant cells that contain two nonsynonymous mutations in exon 7 of *p53 *gene, while Raji cells contain mutations in exon 6 [39]. Nevertheless, the mutated proteins are stabilized in these cells and can accumulate to relatively high levels [39] (and our data not shown). As shown in Figure 6C, Namalwa cells were highly susceptible to DRB-induced death within the 40–100 μM range in a dose-dependent manner (mean 11% viable cells at 72 hr with 100 μM DRB) and the death mechanism was apoptosis, as demonstrated by their positive staining for active caspase-3 and cell surface annexin V expression (mean 75% and 63% of positive cells, respectively, at 24 hr with 100 μM DRB) (Figure 6D). It is noticeable that Namalwa cells were more sensitive at 24 h than control cells. Raji cells were also susceptible to DRB-induced cytotoxicity, albeit with some differences in the death pathway and kinetics: DRB-induced death occurred only after 48 hr (Figure 6E) and apoptosis was not the solely mechanism of death, as caspase-3 activation and cell surface annexin V expression were only detectable after 48 hr and in a low percentage of cells (18% and 27% of positive cells, respectively, at 48 hr with 100 μM DRB) (Figure 6E).

### Blast cells from a primary AML sample are highly sensitive to DRB treatment

**Figure 7:**
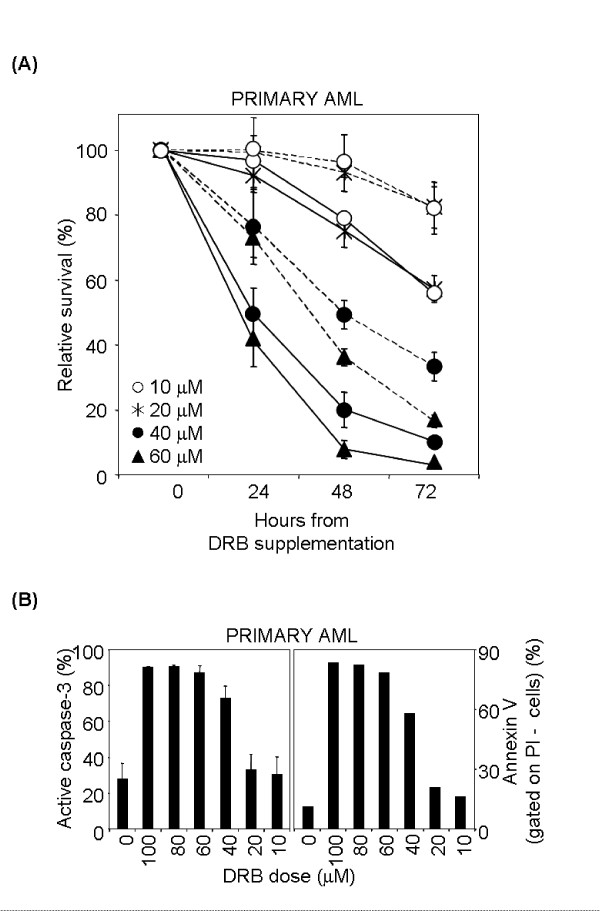
**Sensitivity to DRB-induced apoptosis in primary AML sample**. (A) Differential effect of DRB against tumour versus non-malignant cells. Total nucleated cells separated from the bone marrow of an AML patient were kept in medium alone or containing 10–60 μM DRB, harvested at 24, 48 and 72 hr and analysed by flow cytometry after stainig with anti-CD45 antibody and PI. Blast cells (unbroken line) were selected based on CD45/SSC parameters. Non-malignant cells (broken line) were selected by exclusion of blast cells and debris. Viability of tumour versus non-malignant cells differed in a statistically significant way (p < 0.005 with the test of independence at all doses analysed). (B) DRB treatment induces apoptosis in primary AML cells. Left panel: AML cells were kept for 24 hr in medium alone or containing 10–100 μM DRB, harvested and stained with anti-active caspase-3 antibody (left panel) or annexin V and PI (right panel). Histograms show the percentage of active caspase-3^+ ^cells (mean ± S.D. of two independent experiments) and PI- annexin V+ cells.

Lastly, the effect of DRB on cell viability and apoptosis induction was studied in a human primary AML sample. Leukemic blasts, selected through SSC/CD45 parameters [40] accounted for about 60% of total cells. To define the sensitivity of both tumour blasts and non-malignant cells to this drug, we assessed the viability of treated cells in a dose-response experiment using CD45/PI staining and flow cytometry. Blast cells were susceptible to DRB-induced death within the 10–100 μM range in a dose-dependent manner (mean 3% viable cells at 72 h with = 60 μM DRB; p < 0.005 with the test of independence at all doses, compared to untreated cells) (Figure 7A and data not shown) and the death mechanism was apoptosis (Figure 7B). Interestingly, blast cells were significantly more sensitive to DRB than non-malignant cells at all doses analysed (p < 0.005 with the test of independence).

## Discussion

Here we show that the cyclin-dependent kinase inhibitor DRB efficiently leads normal and leukemic cells to apoptosis in a DNA replication- and DNA damage-independent manner. In T cells expressing wild type p53, DRB induced a cytosolic p53 response that led the cells to apoptosis through Bax activation. Nevertheless, apoptosis was also induced in p53 deficient/mutated leukemic cells through alternative pathways, possibly involving p73. Overall, the transcription stress response imposed by DRB is very powerful in inducing apoptosis independently of a cell's p53 status, and could provide an attractive approach to the treatment of some forms of cancer.

Agents such as DRB that circumvent p53-mediated apoptosis and are not associated with DNA damage may prove valuable in the chemotherapy of both p53-wild type and p53-mutated tumours, with a decreased risk of therapy-related, secondary malignancies. Here we show that prototypic T-, B- and myelogenous leukaemia/lymphomas are all susceptible to DRB-induced death irrespective of their p53 status. Raji cells, which were used as a prototypic example of a chemoresistant Burkitt's lymphoma-derived line [41] that cannot be led to apoptosis by C2-ceramide or other antitumour agents, such as doxorubicin and etoposide [42,43], were efficiently induced to die by DRB. Moreover, the induction of apoptosis in non-proliferative G0/G1 cells inherent in DRB's activity is a very attractive attribute, as quiescence is the major feature of the resistant cells that follow exposure to current chemotherapy. Finally, the evidence obtained in one AML patient of a differential effect of DRB against tumour versus non-malignant cells might suggest that drug toxicity could be minimized by carefully determining the minimal effective dose.

It has been suggested that pol II inhibitors may also prove useful in the treatment of tumours with changes in DNA repair capacity [44-46] and patients with chronic DNA repair deficiency syndromes such as AT and NBS, xeroderma pigmentosum and Cockayne syndrome [3] for which genotoxic treatments are strongly discouraged. The present study demonstrates that the apoptotic pathway induced by DRB is independent of ATM and NBS1, namely the proteins defective in AT and the NBS, respectively. Thus tumours arising in these patients might be efficiently and safely treated with DRB. Moreover, ATM is frequently inactivated in sporadic cancers, particularly lymphoid malignancies [47]. Here, too, DRB could constitute a very convenient therapeutic option.

It could be interesting to evaluate DRB for possible clinical applications. In comparison to other CDK inhibitors already in clinical trials, including flavopiridol and seliciclib, DRB remains one of the most CDK9-selective inhibitors [48]. CDK9 is the kinase with the most specific function limited to regulation of transcription; this selectivity confers the ability to inhibit pol II phosphorylation rather than cell cycle CDKs or other kinases and to target noncycling cancer cells. Moreover, differently from flavopiridol that may intercalate into the DNA thereby damaging it [49], DRB does not induce DNA damage.

Relatively high concentrations of DRB have been used in this work. These could perhaps be reduced by combination with other drugs if DRB were to be proposed for chemotherapy, since combination of CDK inhibitors, including DRB, with the Mdm2 antagonist, nutlin-3a, leads to the synergistic activation of the cytotoxic p53 functions and allows reduction of the concentrations of both drugs [50]. Similar combinations could be suggested to enhance the p53-independent pathways. For example, p53-independent induction of p21Waf1/Cip1 characteristic of histone deacetylase inhibitors [51] resulted into enhanced cytotoxicity when combined with CDK inhibitors [52,53].

Blockade of pol II-dependent transcription triggers a cell death signal [11,13,20,23], though the exact underlying mechanisms were unclear, particularly with respect to its p53-dependence and the need for ongoing DNA replication. Inhibition of pol II (by α-amanitin treatment, RNAi approach, and high dose UV irradiation) has been found to induce p53-dependent apoptosis associated with translocation of p53 to mitochondria, but only upon entry of cells into S phase [11,54] or without entry into S phase, yet in a p53-independent way [13]. Our data help to clarify these issues since we show that in p53-competent cells induction of a transcription-independent cytosolic function of p53 and subsequent Bax activation are the driving forces of DRB-induced apoptosis. This is in accordance with recent data suggesting that blockade of pol II-mediated transcription induced p53 accumulation, and that this is critical for eliciting p53-dependent but transcription-independent apoptosis [54,55]. However, in the absence of p53 DRB efficiently elicits apoptosis through an alternative route that may rely on p73 (see below). We also demonstrate that DRB-induced apoptosis occurs in G0/G1 cells, without entering into S phase and is thus free from significant DNA replication, and that p53 accumulation and susequent apoptosis are independent of possible DNA damage as already reported [20,54]. Our data thus yield new insights into the mechanism of cell death induced by transcriptional blockade.

Some authors have found that inhibition of pol II by inhibitors of the phosphorylation of the pol II CTD, including DRB, resulted in the nuclear accumulation of p53 without concomitant phosphorylation of the Ser15 site of p53 [56,57]. Thus a question was raised as to how p53 was able to accumulate in the nucleus without Ser15 phosphorylation, this being the modification known to maintain p53 in the nuclear fraction. While confirming that the initial accumulation of p53 induced by DRB is not accompanied by Ser15 phosphorylation, our time course determination of p53 localization in normal T cells now clearly shows that p53 is not preferentially accumulated in the nucleus following DRB treatment. Indeed, it is both strongly and rapidly accumulated in their cytosol, as shown by both confocal microscopy analysis and fractionation experiments followed by immunoblotting. As previously described [56], although the initial incubation of p53 is independent of Ser15 phosphorylation, prolonged incubation with DRB induces a secondary stress response leading to Ser15 phosphorylation. However, it appears clear that the initial accumulation of p53 does not require Ser15 phosphorylation and is not related to DNA damage.

The Jurkat T-cell line lacks p53 protein. The absence of a crucial component necessary for the DRB-induced apoptosis of normal human T lymphocytes might have suggested that Jurkat cells were insensitive to DRB. Instead, they proved to be sensitive to DRB and in the present study we begin to explore the mechanism of cell killing in the absence of p53. The rapid (within 1 hour) accumulation of p73 upon treatment of Jurkat cells with DRB suggests that this protein replaces p53 in the induction of apoptosis. p73 is a member of the *p53 *gene family and has been implicated in the apoptosis induced by a variety of chemotherapeutic drugs whose mechanism of action involves DNA damage [58,59]. Our findings in the Jurkat cells provide the first suggestion that activation of pro-apoptotic p73 also couples transcriptional inhibition to apoptosis in keeping with a recent demonstration of the ability of p73 to directly induce cytochrome c release from isolated mitochondria[60]. The role of p73 in mediating DRB-induced apoptosis in the absence of p53 is further supported here by the demonstration that chemical inhibition of p53 activity in a p53 wild-type tumour cell line does induce p73 accumulation.

In our tumour cell lines, and particularly in Raji cells, the mechanism of cell death was not solely apoptosis. Indeed, Raji cells have been described to be apoptosis-resistant due to defects other than p53 mutations, such as impaired apoptotic signal transduction in the cytoplasm [43]. Since DRB apparently induces a death response that overcomes dysregulated apoptosis in these cells, other modes of cell death may be supported to be involved in the DRB-induced response. These remain to be determined, but may include necrosis, mitotic catastrophe and autophagy, as well as premature senescence [61]. DRB also inhibits protein kinases involved in cellular metabolism such as CK1 and CK2 [22]. It is thus possible that cell response following DRB treatment could be influenced by the concomitant inhibition of different kinases.

## Conclusion

In conclusion, our results yield new insight into the apoptotic pathway induced by DRB and suggest that it could be used to treat some forms of cancer on account of its striking ability to induce cell death irrespective of p53 status and without genotoxic stress.

In this paper, we yield new insight into the apoptotic pathway induced DRB, an inhibitor of the transcriptional CDKs 7 and 9. Prototypic T-, B- and myelogenous leukaemia cell lines as well as fresh AML blasts were all susceptible to DRB-induced cell death, suggesting that the transcription stress response imposed by DRB is very powerful in inducing apoptosis independently of a cell's p53 status and without inducing DNA damage, thus providing an attractive approach to the treatment of some forms of cancer.

## Competing interests

The authors declare that they have no competing interests.

## Authors' contributions

VT carried out all the experiments. PP and LO participated in the data analysis and provided technical support. MD and AA provided critical revision of the manuscript for important intellectual content. CG and VT designed the study and CG coordinated this work. All authors significantly contributed to data interpretation and manuscript drafts. All authors read and approved the final version of the manuscript.

## Pre-publication history

The pre-publication history for this paper can be accessed here:

http://www.biomedcentral.com/1471-2407/9/281/prepub
